# Non-cirrhotic portal vein thrombosis – therapeutic challenge

**DOI:** 10.1590/1677-5449.210013

**Published:** 2022-03-07

**Authors:** Lucas Mansano Sarquis, Paula de Oliveira Trintinalha, Wilson Michaelis, Antonio Lacerda Santos, Rogerio Akira Yokoyama, Thiago Michaelis, Adriana Pires Smaniotto, Mariana Santos Oliveira

**Affiliations:** 1 Hospital do Trabalhador – HT, Curitiba, PR, Brasil.; 2 Hospital Universitário Evangélico Mackenzie – HUEM, Curitiba, PR, Brasil.

**Keywords:** embolism and thrombosis, mesenteric vein thrombosis, anticoagulants

## Abstract

Portal vein thrombosis (PVT) is a disease in which thrombosis occurs from the intrahepatic branches of the portal vein, and may extend to the splenic vein and/or superior mesenteric vein. It is most often associated with liver cirrhosis. PVT not associated with cirrhosis is rare. The aim of this article is to report two cases of PVT in which it was not associated with cirrhosis. Both were treated with anticoagulation and clinical progress afterwards was good.

## INTRODUCTION

Portal vein thrombosis (PVT) is a disease in which thrombosis occurs from the intrahepatic branches of the portal vein as far as the splenic vein and/or superior mesenteric vein.^1^ There is no single etiology responsible for PVT, which can be related to many different factors, such as hereditary or acquired thrombophilias, cancer, liver disease and/or cirrhosis, local inflammatory damage, and/or portal system injuries. Prevalence can reach 1% in the general population and is higher among people with liver disease.^2^

The clinical presentation of PVT may be acute (< 60 days) or chronic^3^ and it is not always easy to differentiate between the two forms. Clinical manifestations related to PVT can include abdominal pains involving the right hypochondrium, splenomegaly, ascites, or fever, but it can also manifest asymptomatically and be identified by imaging exam findings.^3^ The gold standard for diagnosis is computed tomography angiography (CTA) or magnetic resonance imaging (MRI) of the abdomen, which, in addition to diagnosing PVT, can also rule out comorbidities such as malignant tumors or intestinal infarctions. Additional work up with laboratory tests, such as those for markers of genetic or acquired thrombophilias, can be useful to confirm diagnosis.^4^

Once any need for urgent surgery has been ruled out, treatment is initiated with full anticoagulation, whether using intravenous unfractionated heparin (UFH) or subcutaneous low molecular weight heparin (LMWH), which are both equally effective.^5^ After stabilization of clinical status, patients can be followed-up in outpatients and anticoagulation is recommended with the objective of averting progression of the PVT.^5^

The objective of this article was to describe two cases of PVT unrelated to cirrhosis that were treated with anticoagulation and exhibited satisfactory clinical progress.

The study protocol was approved by the Ethics Committee at our institution (CAAE 38798220.1.0000.0103, decision number 4.350.712).

## PART I - CLINICAL SITUATION

### Case 1

The patient was a 54-year-old woman who was admitted via the emergency room to a tertiary hospital in Curitiba, Paraná, Brazil, after presenting with diffuse abdominal pains with onset 2 days earlier that had worsened progressively, but with no nausea or vomiting. Physical examination found her vital signs to be stable, digestive sounds present, with a mildly distended abdomen, mild diffuse pain on palpation, and no signs of peritoneal irritation. She was an ex-smoker with hypothyroidism, was taking levothyroxine, and was on tibolone hormone replacement therapy. She stated that she had never had abdominal surgery previously.

**Figure 1:**
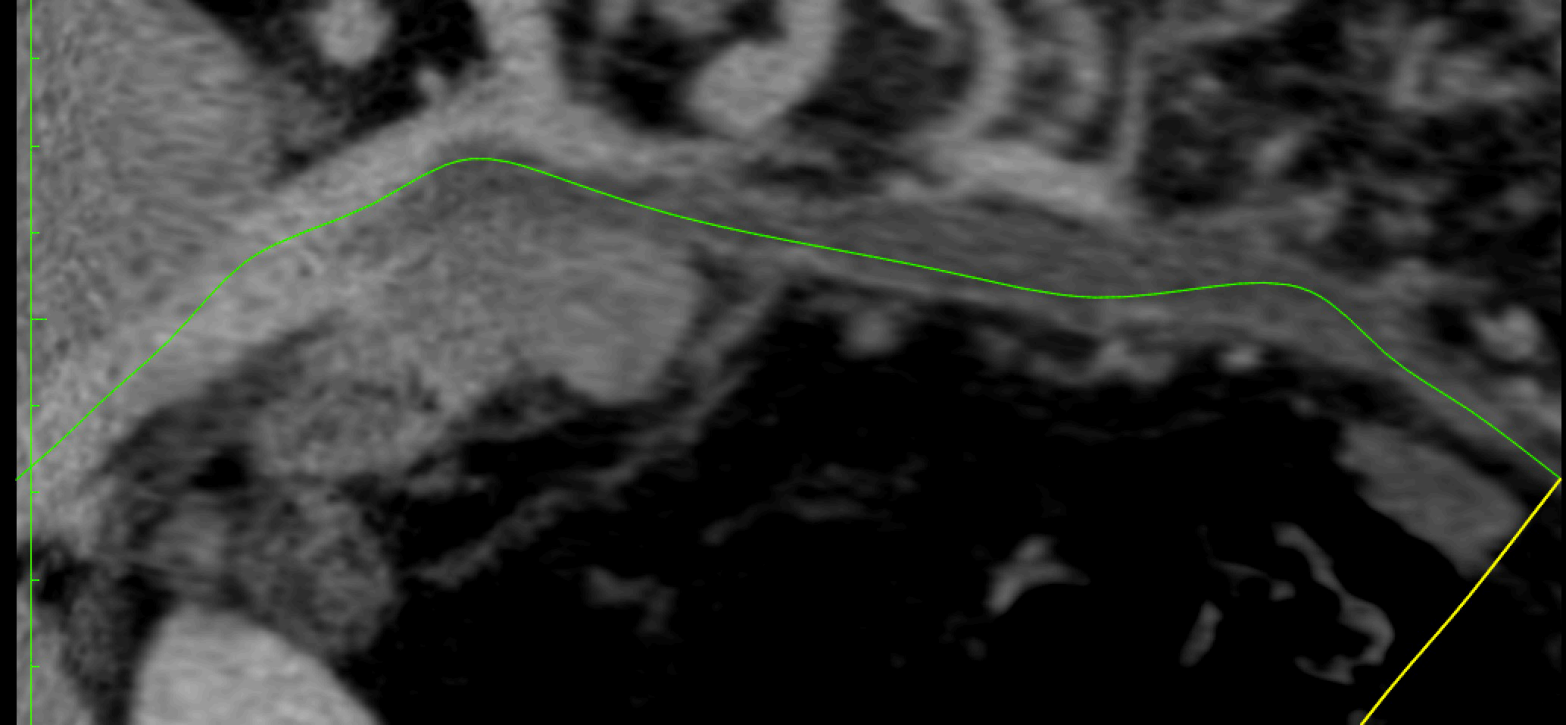
Computed tomography angiography of the abdomen and pelvis, showing images suggestive of failure to fill the interior of portal and superior mesenteric veins, compatible with presence of thrombi.

**Figure 2:**
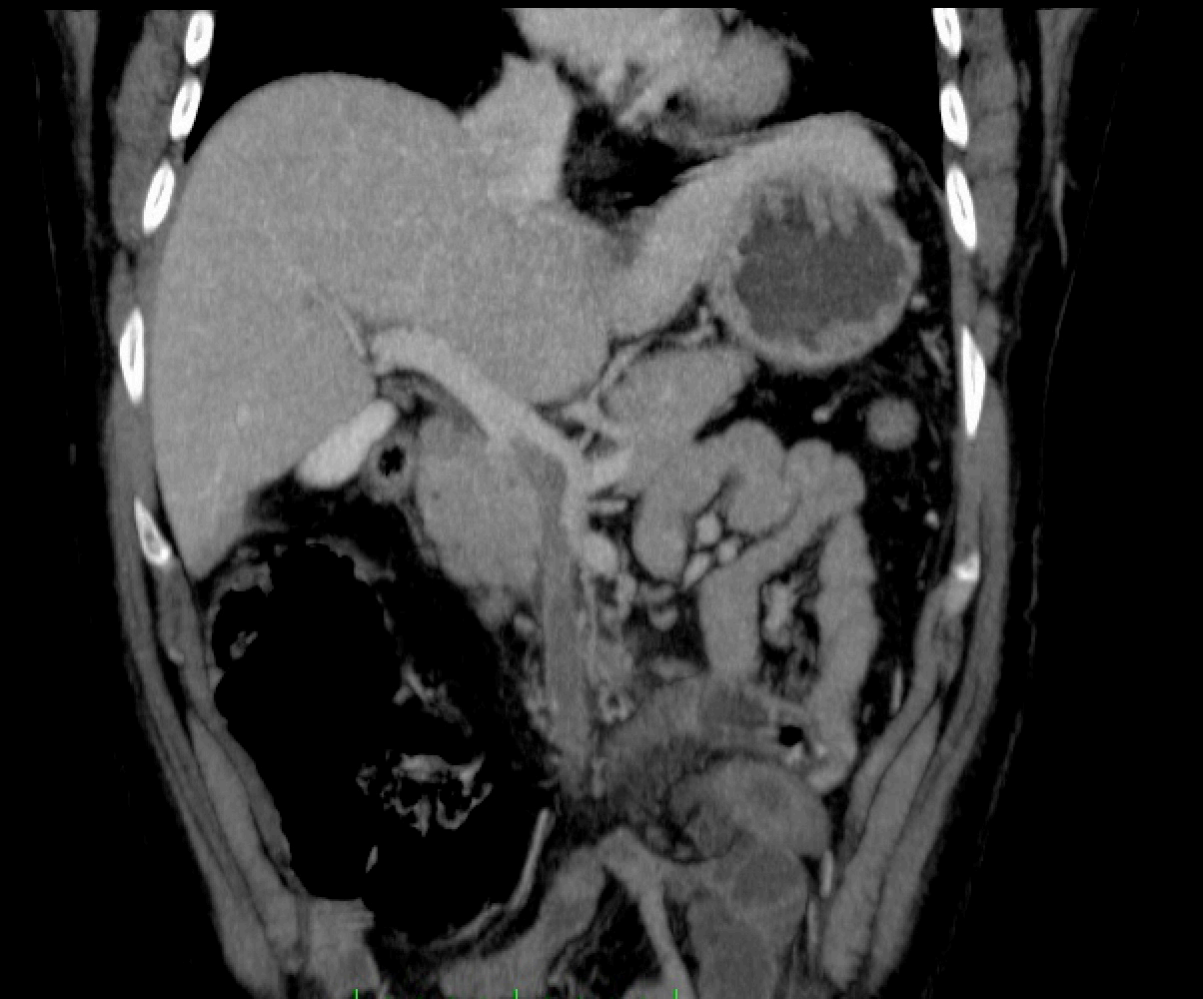
Computed tomography angiography of the abdomen and pelvis, showing images suggestive of failure to fill the interior of portal and superior mesenteric veins, compatible with presence of thrombi..

She underwent CTA of the abdomen/pelvis (Figures 1 and 2), which showed evidence of failure to fill the portal and superior mesenteric veins, with images compatible with thrombi, and also edema of small intestine loops, with venous engorgement and increased fat density in the adjacent mesentery.

### Case 2

The patient was a 72-year-old man seen at the clinic, where he had presented complaining of nonspecific abdominal pains for the preceding 2 weeks, with no other associated symptoms. Physical examination found his vital signs stable, his abdomen flaccid, but painful on palpation, and no signs of peritonitis. He did not smoke. His comorbidities were coronary artery disease, systemic arterial hypertension, dyslipidemia, obesity, and diabetes mellitus type II. He was taking acetylsalicylic acid, oral antiglycemics, and valsartan. He stated that he never had abdominal surgery previously.

**Figure 3:**
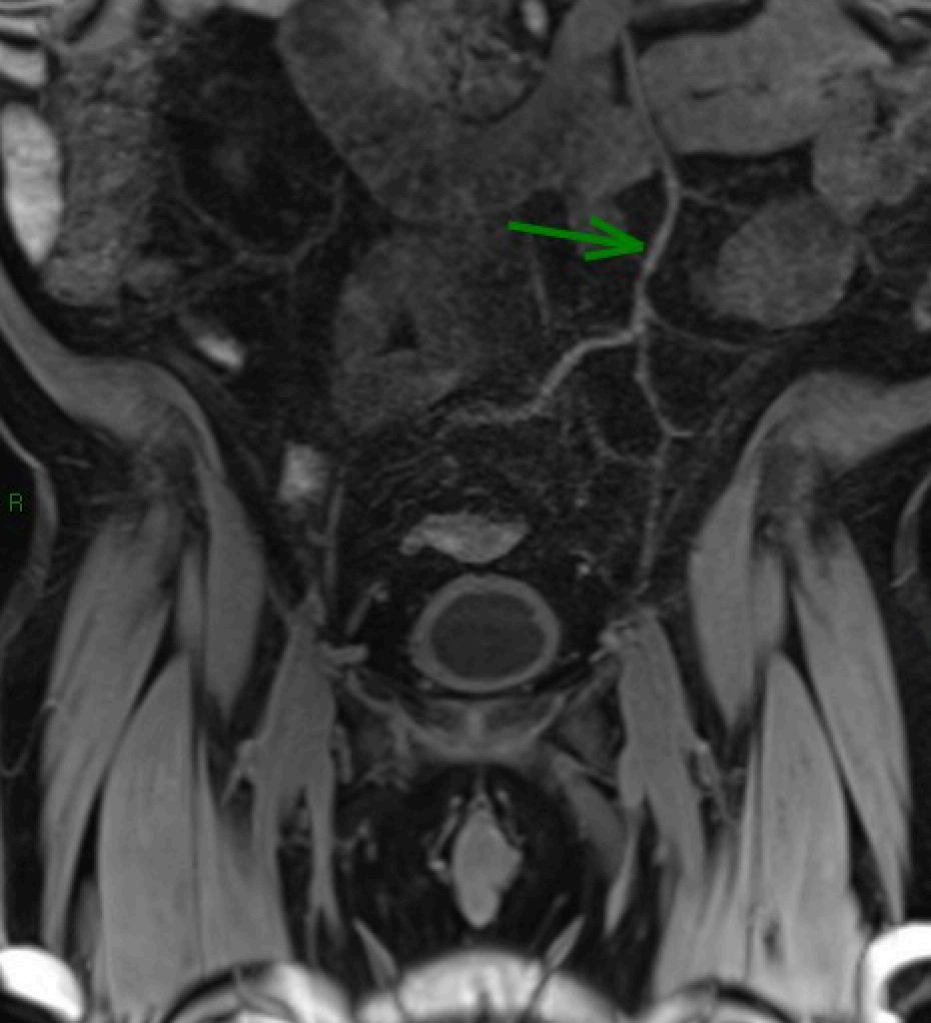
Magnetic resonance angiography of the abdomen and pelvis, as described in case 2, showing images suggestive of apparently acute thrombosis of the superior mesenteric vein and a segment of the portal vein.

The patient underwent MRI of the abdomen and pelvis (Figure 3), which showed images suggestive of thrombosis, apparently acute, of the superior mesenteric vein and a segment of the portal vein, in addition to slightly elevated fat density around the thrombosed segments.

## PART II - WHAT WAS DONE

### Case 1

In view of the signs of the uncertainty with regard to presence of a small intestine tumor, the decision was taken to conduct an explorative laparotomy, during which thickening of intestinal loops was observed, but no evidence of a tumor was seen. Tests for neoplasms conducted before starting anticoagulation had been negative. Anticoagulation was initiated on the first postoperative day with 60 mg of subcutaneous enoxaparin, repeated every 12 hours for 8 days.

**Figure 4:**
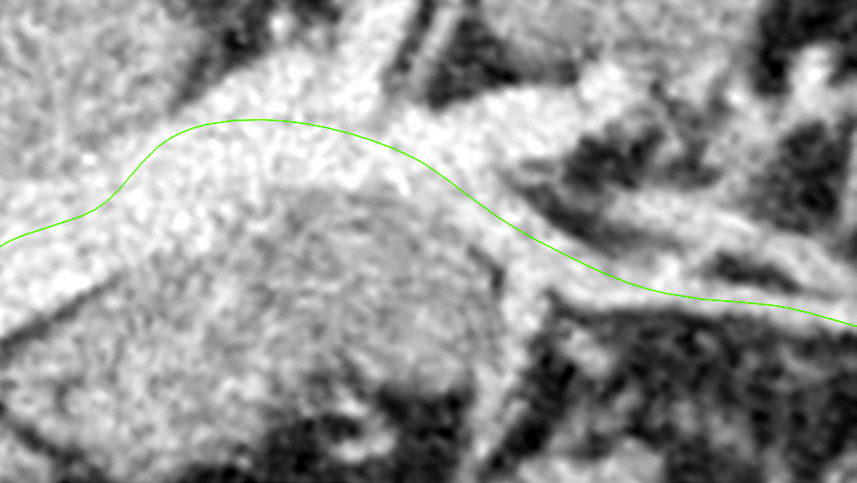
Computed tomography angiography of the abdomen and pelvis 6 months after treatment with anticoagulation, showing no filling failures – case 1.

After observing good clinical progress, the patient was discharged from hospital on 15 mg of rivaroxaban every 12 hours, for 21 days, after which she was put on 20 mg of rivaroxaban per day. Six months after diagnosis, control CTA of the abdomen/pelvis (Figure 4) revealed no further failures to fill the visceral veins. The patient is currently asymptomatic and in outpatients follow-up.

### Case 2

After collecting samples to test for thrombophilias and neoplasms, anticoagulation was initiated with 60 mg of subcutaneous enoxaparin, repeated every 12 hours for 6 days.

The patient was discharged from hospital on 10 mg of apixaban every 12 hours, for 7 days and then 5 mg of apixaban every 12 hours thereafter. Platelet antiaggregation was maintained because of his underlying cardiac disease.

**Figure 5:**
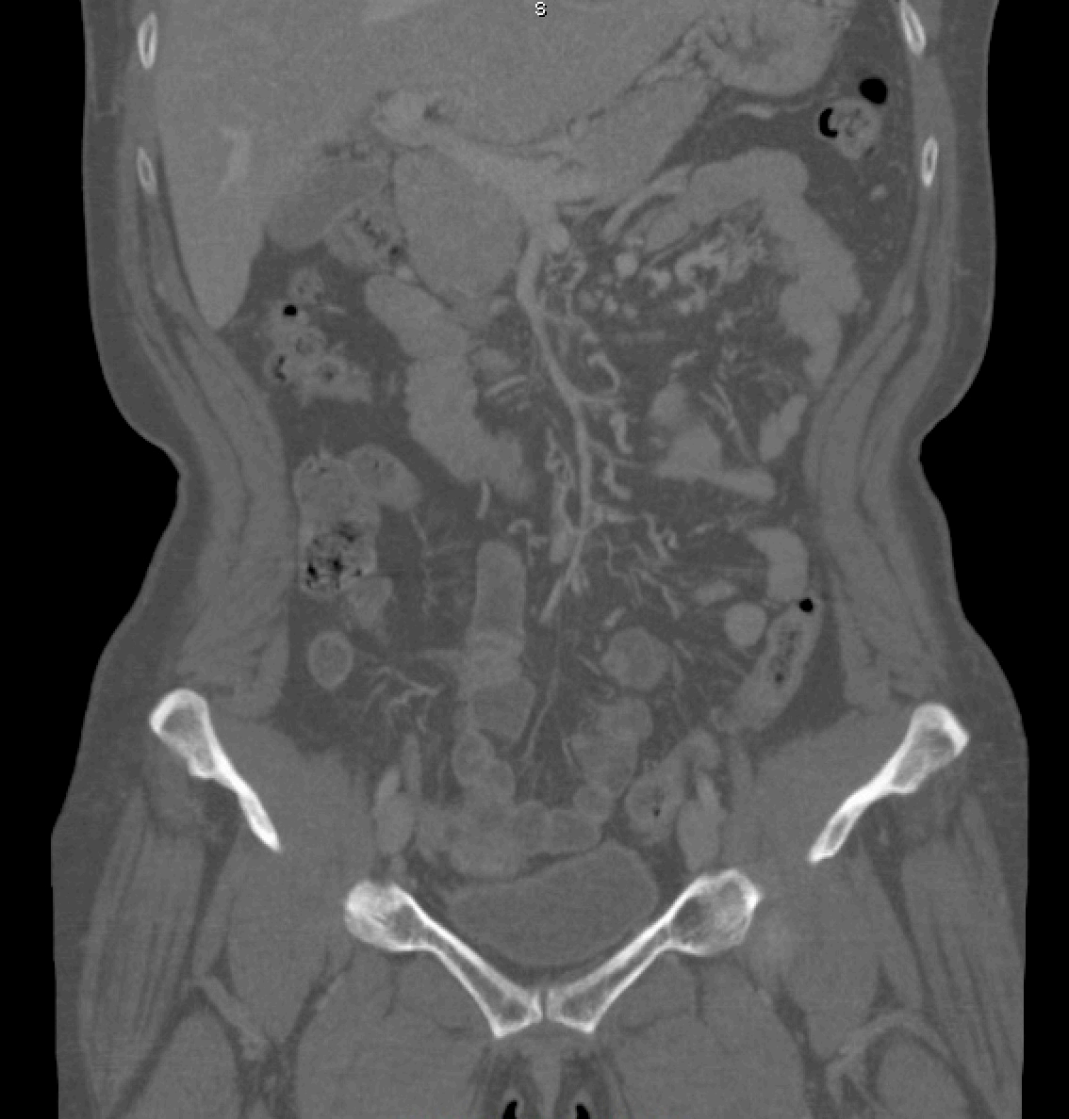
Computed tomography angiography of the abdomen and pelvis 6 months after treatment with anticoagulation, showing no filling failures– case 2.

Six months after diagnosis, control CTA of the abdomen/pelvis (Figure 5) revealed no further failures to fill the visceral veins. The patient is currently asymptomatic and in outpatients follow-up.

## DISCUSSION

Symptoms comprising nonspecific abdominal pains may be the initial clinical manifestation, as seen in these two reports. Absence of hepatic disease and/or neoplasms may delay clinical diagnosis of PVT. There are many different risk factors related to this disease: genetic or acquired thrombophilia, cancer (in particular hepatocarcinomas), abdominal inflammations (pancreatitis, cholecystitis, appendicitis), and abdominal surgeries (splenectomy, colectomy).^4^ Hormone replacement therapy with estrogens can also be a trigger factor in development of PVT, as seen in case 1.^6^ However, PVT can also develop even in the absence of identifiable risk factors, as seen in case 2.

Since the initial clinical manifestation in the majority of cases is abdominal pains, investigation can be started with abdominal ultrasonography, to rule out other etiologies of PVT. In turn, CTA is a rapid examination that is available at the majority of tertiary hospitals in Brazil and can confirm or rule out other differential diagnoses.^5^ The contrast used for CTA must be administered intravenously, since contrast given orally can interfere with interpretation of the images. A multidisciplinary approach is essential for good management of the condition and good clinical progress, and the need for surgery with explorative laparotomy to investigate peritonitis and abdominal pains seen in case 1 underscores its importance.

The principal thrombophilias that should be investigated are factor V Leiden mutation, antithrombin deficiency, C and S protein deficiencies, prothrombin gene mutations, and antiphospholipid antibody syndrome.^3^ It should be remembered that tests for these thrombophilias can be affected by anticoagulation. It is important to note that presence or absence of thrombophilias does not change the indication of anticoagulation in the acute phase, which should be chosen on a case-by-case basis. However, these tests are a useful support for clinical decision-making and can influence the extent of anticoagulation and help determine the choice of anticoagulant to be prescribed. Thrombophilia tests were negative in both patients described here.

Anticoagulant treatment is indicated to avert progression of the PVT until its etiology can be identified. There is no evidence that differentiates between the efficacy or safety of different anticoagulants for treatment of PVT. However, despite the scant scientific evidence in that respect, direct oral anticoagulants (DOACs) tend to be the preferred choice because of their simpler posology.^7^^,^ 8

The duration of anticoagulation should be at least 3 to 6 months, or for as long as the risk factors are present. However, DOACs have some limitations: the elevated price, the need to adjust the dose in the presence of renal failure, and the lack of any agents available in Brazil to reverse factor X inhibitor DOACs.^9^ Use of DOACs appears to be a safe and viable option in cases unrelated to cirrhosis or those related to hormone replacement,^6^^,^ 10 ^,^ 11 ^,^ 12 as in case 1.

There is still limited evidence for alternative treatments, such as surgical thromboembolectomy, systemic or in situ thrombolysis, or transjugular intrahepatic portosystemic shunt (TIPS), which involve intrinsic risks and are not associated with improved morbidity or mortality.^13^ For relapses associated with advanced liver cirrhosis, liver transplantation may be the last treatment option.^5^

Treatment of PVT in asymptomatic patients remains controversial. The tendency is to indicate anticoagulation, but always after assessing the balance of risk and benefit between the chance of bleeding and avoiding relapse. Watchful waiting is one possible option for patients with PVT caused by transitory risk factors (intra-abdominal inflammatory processes).^6^

The principal complications related to chronic PVT are persistent chronic abdominal pains and gastrointestinal bleeding. However, the risk of bleeding is more related to the underlying disease than to use of DOACs.^7^ Progression of PVT may compromise multiple intra-abdominal organs, which can constitute indications for more aggressive treatments and may even require urgent multiple visceral transplants in specific cases.^14^

## CONCLUSIONS

Portal vein thrombosis is a complex disease that demands a multidisciplinary approach to ensure better understanding and treatment. Computed tomography angiography is now the gold standard examination for diagnosis and for assessing progression of thrombosis. Anticoagulation with DOACs appears to be a safe and effective form of treatment for these patients, but it is always necessary to weigh up the risks of bleeding. There is a clear need for prospective, randomized, and multicenter studies to evaluate the best approach to management of patients with PVT unrelated to cirrhosis.

## References

[1] Chawla YK, Bodh V (2015). Portal vein thrombosis. J Clin Exp Hepatol.

[2] Qi X (2017). Portal vein thrombosis: recent advance. Adv Exp Med Biol.

[3] Hanafy AS, Abd-Elsalam S, Dawoud MM (2019). Randomized controlled trial of rivaroxaban versus warfarin in the management of acute non-neoplastic portal vein thrombosis. Vascul Pharmacol.

[4] Salembier A, Verhamme M, Verhamme P, Van Moerkercke W (2018). Acute non-cirrhotic portal vein thrombosis: review. Acta Gastroenterol Belg.

[5] Sabol TP, Molina M, Wu GY (2015). Thrombotic venous diseases of the liver. J Clin Transl Hepatol.

[6] Sharma AM, Zhu D, Henry Z (2016). Portal vein thrombosis: when to treat and how?. Vasc Med.

[7] Intagliata NM, Caldwell SH, Tripodi A (2019). Diagnosis, development, and treatment of portal vein thrombosis in patients with and without cirrhosis. Gastroenterology.

[8] Turon F, Hernández-Gea V, García-Pagán JC (2018). Portal vein thrombosis: yes or no on anticoagulation therapy. Curr Opin Organ Transplant.

[9] Priyanka P, Kupec JT, Krafft M, Shah NA, Reynolds GJ (2018). Newer oral anticoagulants in the treatment of acute portal vein thrombosis in patients with and without cirrhosis. Int J Hepatol.

[10] Ghazaleh S, Beran A, Aburayyan K (2021). Efficacy and safety of anticoagulation in non-malignant portal vein thrombosis in patients with liver cirrhosis: a systematic review and meta-analysis. Ann Gastroenterol.

[11] Loffredo L, Pastori D, Farcomeni A, Violi F (2017). Effects of Anticoagulants in Patients With Cirrhosis and Portal Vein Thrombosis: A Systematic Review and Meta-analysis. Gastroenterology.

[12] Mohan BP, Aravamudan VM, Khan SR, Ponnada S, Asokkumar R, Adler DG (2020). Treatment response and bleeding events associated with anticoagulant therapy of portal vein thrombosis in cirrhotic patients: systematic review and meta-analysis. Ann Gastroenterol.

[13] Shatzel JJ, O’Donnell M, Olson SR (2019). Venous thrombosis in unusual sites: a practical review for the hematologist. Eur J Haematol.

[14] Vianna R, Beduschi T (2016). Multivisceral transplantation for diffuse splanchnic venous thrombosis. Curr Opin Organ Transplant.

