# Supplementary material for: Non-cirrhotic portal vein thrombosis – therapeutic challenge
Source: J Vasc Bras. 2022 Mar 7;21:e20210013. doi: 10.1590/1677-5449.210013 (PMC8958433; doi:10.1590/1677-5449.210013)
Supplement: Translation

# Translation: Trombose de veia porta não associada à cirrose – desafio terapêutico

**Authors:** Lucas Mansano Sarquis, Paula de Oliveira Trintinalha, Wilson Michaelis, Antonio Lacerda Santos, Rogerio Akira Yokoyama, Thiago Michaelis, Adriana Pires Smaniotto, Mariana Santos Oliveira

**Affiliations:** 1 Hospital do Trabalhador – HT, Curitiba, PR, Brasil.; 2 Hospital Universitário Evangélico Mackenzie – HUEM, Curitiba, PR, Brasil.

**Keywords:** embolia e trombose, trombose venosa mesentérica, anticoagulantes

## Abstract

A trombose de veia porta (TVP) é uma doença na qual ocorre trombose desde os ramos intra-hepáticos da veia porta, podendo se estender até a veia esplênica e/ou veia mesentérica superior, estando associada, na maioria das vezes, à cirrose hepática. A TVP não associada a cirrose é rara. O objetivo deste artigo foi relatar dois casos de TVP não associados à cirrose, que foram tratados com anticoagulação e tiveram evolução clínica satisfatória.

## INTRODUÇÃO

A trombose de veia porta (TVP) é uma doença na qual ocorre trombose desde os ramos intra-hepáticos da veia porta até a veia esplênica e/ou veia mesentérica superior^1^. Não há uma etiologia única para a TVP, podendo estar relacionada a diversos fatores, como trombofilias hereditárias ou adquiridas, câncer, doença hepática e/ou cirrose, lesões inflamatórias locais e/ou lesão do sistema portal. A prevalência pode chegar até 1% na população em geral, sendo maior nos portadores de doença hepática^2^.

A apresentação clínica da TVP pode se dar de forma aguda (< 60 dias) ou crônica^3^. A diferenciação entre as duas formas nem sempre é fácil. As manifestações clínicas relacionadas podem ser dor abdominal em hipocôndrio direito, esplenomegalia, ascite ou febre, podendo, ainda, ser manifestada de forma assintomática como achado de exame de imagem^3^. O diagnóstico padrão-ouro é realizado através da angiotomografia computadorizada (ATC) ou da ressonância nuclear magnética (RNM) de abdome, que, além de diagnosticar a TVP, pode descartar comorbidades, como lesões malignas associadas ou infartos intestinais. A complementação com exames laboratoriais, como marcadores de trombofilias genéticas ou adquiridas, pode ser útil para elucidação diagnóstica^4^.

Após descartar a necessidade de cirurgia de urgência, o tratamento inicia-se com a anticoagulação plena, seja com heparina não fracionada (HNF) endovenosa ou heparina de baixo peso molecular (HBPM) subcutânea, sendo ambas igualmente efetivas^5^. Após estabilização do quadro clínico, é possível realizar seguimento ambulatorial desses pacientes, sendo preconizada a anticoagulação com intuito de evitar a progressão da TVP^5^.

O objetivo deste artigo foi relatar dois casos de TVP não associados à cirrose, que foram tratados com anticoagulação e tiveram evolução clínica satisfatória.

O protocolo foi aprovado pelo Comitê de Ética de nossa instituição (CAAE 38798220.1.0000.0103, parecer número 4.350.712).

## PARTE I – SITUAÇÃO CLÍNICA

### Caso 1

Paciente do sexo feminino, 54 anos de idade, admitida no serviço de emergência de um hospital terciário em Curitiba, Paraná, com quadro de dor abdominal difusa há dois dias com piora progressiva, sem náuseas ou vômitos associados. Ao exame físico, encontrava-se com sinais vitais estáveis, abdome com ruídos hidroaéreos presentes, levemente distendido, dor leve à palpação difusa e sem sinais de irritação peritoneal. Ex-tabagista, portadora de hipotireoidismo, em uso de levotiroxina e terapia de reposição hormonal com tibolona. Nega cirurgias abdominais prévias.

Realizou-se ATC de abdome/pelve (Figuras 1 e 2), sendo evidenciada falha de enchimento no interior das veias porta e mesentérica superior, compatíveis com imagens de trombos, além de edema de alças de intestino delgado com ingurgitamento venoso e densificação da gordura do mesentério adjacente.

**Figura 1:** Angiotomografia computadorizada de abdome e pelve, com imagem sugestiva de falha de enchimento no interior das veias porta e mesentérica superior, compatíveis com a presença de trombos.

**Figura 2:** Angiotomografia computadorizada de abdome e pelve, com imagem sugestiva de falha de enchimento no interior das veias porta e mesentérica superior, compatíveis com a presença de trombos.

### Caso 2

Paciente do sexo masculino, 72 anos de idade, atendido no ambulatório, com queixa de dor abdominal inespecífica há 2 semanas, sem outros sintomas associados. Ao exame físico, encontrava-se com sinais vitais estáveis, abdome flácido, doloroso à palpação e sem sinais de peritonite. Negava tabagismo. Apresentava como comorbidades doença arterial coronariana, hipertensão arterial sistêmica, dislipidemia, obesidade e diabetes melito tipo II. Referia o uso de ácido acetilsalicílico, terapia antiglicemiante oral e valsartana. Negava cirurgias abdominais prévias.

O paciente foi submetido a RNM de abdome e pelve (Figura 3), que evidenciou imagem sugestiva de trombose de aspecto agudo da veia mesentérica superior e de segmento da veia porta, além de leve densificação da gordura circunjacente aos segmentos trombosados.

**Figura 3:** Angioressonância de abdome e pelve descrita no caso 2, identificando imagens sugestivas de trombose com aspecto agudo da veia mesentérica superior, segmento da veia porta e da veia mesentérica superior.

## PARTE II – O QUE FOI FEITO

### Caso 1

Devido à incerteza quanto à presença de tumor de intestino delgado, optou-se pela realização de laparotomia exploradora, sendo observado espessamento de alças intestinais, mas sem evidência de tumor. Os exames realizados antes de iniciar a anticoagulação para investigação de neoplasia e trombofilias foram negativos. No primeiro dia do pós-operatório, foi iniciada anticoagulação com enoxaparina 60 mg via subcutânea, de 12 em 12 horas, por 8 dias.

Após boa evolução clínica, foi dada alta hospitalar à paciente, em uso de rivaroxabana 15 mg, de 12 em 12 horas por 21 dias; após esse período, iniciou-se rivaroxabana 20 mg por dia. Realizou-se ATC de abdome/pelve de controle (Figura 4) após 6 meses do diagnóstico, que não identificou presença de falhas de enchimento em veias viscerais. Atualmente, a paciente encontra-se assintomática e em seguimento ambulatorial.

**Figura 4:** Angiotomografia computadorizada de abdome e pelve 6 meses após o tratamento com anticoagulação, não identificando falhas de enchimento – caso 1.

### Caso 2

Após a coleta para exames de investigação para trombofilias e neoplasias, a anticoagulação foi iniciada com enoxaparina 60 mg via subcutânea, de 12 em 12 horas, por 6 dias.

O paciente recebeu alta hospitalar com apixabana 10 mg de 12 em 12 horas, por 7 dias, seguido de apixabana 5 mg, de 12 em 12 horas. A antiagregação plaquetária foi mantida por conta da doença cardiológica de base.

Foi realizada ATC de abdome e pelve de controle (Figura 5) após 6 meses do diagnóstico, que não identificou presença de falhas de enchimento em veias viscerais. Atualmente, o paciente encontra-se assintomático e em seguimento ambulatorial.

**Figura 5:** Angiotomografia computadorizada de abdome e pelve 6 meses após o tratamento com anticoagulação, não identificando falhas de enchimento – caso 2.

## DISCUSSÃO

O sintoma inespecífico de dor abdominal pode ser a manifestação clínica inicial, como apresentado por estes dois relatos. A ausência de doenças hepáticas e/ou neoplasias pode atrasar o diagnóstico clínico da TVP. São muitos os fatores de risco associados a essa doença: trombofilia genética ou adquirida, câncer (em especial o hepatocarcinoma), inflamações abdominais (pancreatite, colecistite, apendicite), cirurgias abdominais (esplenectomia, colectomia)^4^. O uso de reposição hormonal com estrógenos pode ser um fator desencadeante para o desenvolvimento da TVP, como apresentado no caso 1^6^. Entretanto, a TVP pode se desenvolver mesmo que não exista fator de risco identificável, como relatado no caso 2.

Como na maioria das vezes a manifestação clínica é dor abdominal, a investigação pode ser iniciada por uma ultrassonografia abdominal, para descartar outras etiologias de TVP. A ATC é um exame rápido, disponível na maioria dos hospitais terciários brasileiros, que pode confirmar ou descartar outros diagnósticos diferenciais^5^. O contraste utilizado para ATC deve ser administrado por via endovenosa, uma vez que o contraste por via oral pode atrapalhar a interpretação das imagens. A abordagem multidisciplinar é essencial para o bom manejo e evolução dessa condição clínica, e a necessidade de abordagem cirúrgica com laparotomia exploradora, para investigação da peritonite e dor abdominal conforme apresentado no caso 1, enfatiza essa importância.

As principais trombofilias que devem ser investigadas são mutação do fator V Leiden, deficiência de antitrombina, deficiência de proteínas C e S, mutação do gene da protrombina e síndrome do anticorpo antifosfolipide^3^. Deve-se ficar atento que a investigação dessas trombofilias pode estar alterada pela anticoagulação. Vale ressaltar que a presença ou ausência das trombofilias não irá mudar a indicação da anticoagulação na fase aguda, devendo-se avaliar, a cada caso, como será feita. Entretanto, esses exames são úteis para complementar o raciocínio clínico, bem como para poder influenciar na extensão de anticoagulação e determinar a escolha do anticoagulante a ser prescrito. A pesquisa de trombofilias foi negativa para ambos os pacientes.

O tratamento anticoagulante está indicado para evitar a progressão da TVP até que se possa confirmar a sua etiologia. Não existem evidências para diferenciar a eficácia e segurança dos diversos anticoagulantes no tratamento da TVP. No entanto, a despeito de pouca evidência científica, os anticoagulantes orais diretos (DOACs) tendem a ser a preferência na escolha, pela facilidade da posologia^7^^,^ 8.

A duração da anticoagulação deve perdurar por no mínimo 3 a 6 meses, ou ainda enquanto os fatores de riscos estiverem presentes. Entretanto, os DOACs apresentam algumas limitações: preço elevado, necessidade de ajuste de dose na insuficiência renal e indisponibilidade no Brasil de agentes de reversão para os DOACs inibidores do fator X ativado^9^. O uso dos DOACs parece ser uma alternativa segura e viável para os casos não relacionados à cirrose ou para aqueles relacionados com reposição hormonal^6^^,^ 10 ^,^ 11 ^,^ 12, como no caso 1.

Tratamentos alternativos, como tromboembolectomia cirúrgica, trombólise sistêmica ou *in situ* ou TIPS (*shunt* portossistêmico intra-hepático via transjugular), permanecem com evidências limitadas e com riscos intrínsecos sem associação com a melhora da morbimortalidade^13^. Para recidivas associadas à cirrose hepática avançada, o transplante hepático pode ser a última alternativa de tratamento^5^.

O tratamento da TVP em pacientes assintomáticos permanece controverso. A tendência é indicar a anticoagulação, porém, sempre avaliando risco-benefício entre evitar a recidiva e a ocorrência de sangramento. O tratamento expectante pode ser uma alternativa para pacientes com TVP causada por fatores de risco não persistentes (processo inflamatório intra-abdominal)^6^.

As principais complicações relacionadas com a TVP crônica são dor abdominal crônica persistente e sangramento gastrointestinal. Entretanto, o risco de sangramento está relacionado mais com a doença de base do que com o uso dos DOACs^7^. A progressão da TVP pode levar ao comprometimento de múltiplos órgãos intra-abdominais, podendo ser indicação de tratamentos mais agressivos e até transplante multivisceral de urgência em casos específicos^14^.

## CONCLUSÃO

A TVP é uma doença complexa, que necessita de abordagem multidisciplinar para melhor compreensão e tratamento. A ATC tornou-se o exame padrão-ouro para diagnosticar e avaliar a progressão da trombose. A anticoagulação com DOACs parece ser a uma forma segura e efetiva para tratamento dos pacientes, sempre devendo-se ponderar os riscos de sangramento. É notória a necessidade de estudos prospectivos, randomizados e multicêntricos para avaliar a melhor conduta nos pacientes com TVP não associada à cirrose.
